# Could Hyperbaric Oxygen Be a Solution in the Treatment of Spinal Infections?

**DOI:** 10.3390/medicina55050164

**Published:** 2019-05-20

**Authors:** Şefika Körpınar

**Affiliations:** Department of Underwater and Hyperbaric Medicine, Faculty of Medicine, Canakkale Onsekiz Mart University, 17000 Canakkale, Turkey; sefikkorpinar@yahoo.com or s_korpinar@yahoo.com

**Keywords:** hyperbaric oxygen, pyogenic spinal infection, spondylodiscitis

## Abstract

*Background and Objective:* Pyogenic spinal infections are rare and potentially devastating, requiring prompt recognition and management. Parallel to the ever-increasing number of invasive spinal procedures, its incidence is on a steady rise, particularly in an expanding elderly population. The aim of this study was to evaluate the efficacy of hyperbaric oxygen (HBO_2_) therapy in the treatment of this heterogeneous group of disorders. *Materials and Methods:* Nineteen patients who were referred to our center for HBO_2_ with a clinical diagnosis of spinal infections (vertebral osteomyelitis, pyogenic spondylitis, spondylodiscitis, surgical site infection following spine surgery, epidural abscess) were retrospectively reviewed. *Results:* Infection resolution was adequately achieved in 12 of 13 patients (92.3%) on magnetic resonance imaging at the end of HBO_2_ treatment or during the first month of follow-up. The mean follow-up period was 11 months (range 1 month to 3 years). *Conclusions:* This study suggests that HBO_2_ therapy is efficacious in patients with pyogenic spinal infections complicated by primary therapy failure or by medical comorbidities that may impede the eradication of microbial infection and delay wound healing. HBO_2_ therapy may be useful for reducing long hospital stays, repeated surgeries, and morbidities.

## 1. Introduction

Spinal infections with involvement of the vertebral body, intervertebral disc, and/or adjacent paraspinal tissue constitute 2–7% of all musculoskeletal system infections [1]. Infection of the vertebral column was first described by Hippocrates, who was regarded as the father of spinal surgery. Until 1779, when Percivall Pott made the first detailed description of tuberculosis infection, which can affect the spine and cause serious damage to the intervertebral discs, little knowledge was added to the basic school of Hippocratic understandings. A century later, Odilon M. Lannelongue described pyogenic osteomyelitis as we recognize it today. With the introduction of new diagnostic methods, therapeutic modalities, and sophisticated instrumentation, the actual progression of pyogenic spinal infections has been recorded over the last century. Parallel to the ever-increasing number of invasive spinal procedures, discitis (or postprocedural disc space infections) were first described following these procedures as a clinical entity by Turnbull in 1953 [2,3,4]. During the past decade, this clinical entity has been on a steady rise with the widespread use of advanced imaging modalities, particularly in an expanding elderly population with substantial comorbidities subject to frequent invasive procedures. It can be said that one of the most significant changes related to pyogenic spinal infections in the literature is undoubtedly incidence data. In recent studies, the figures range from 5.8 to 7.4/100,000 in different series [5,6,7].

The key principles for the successful treatment of spinal infections are antibiotic therapy for the eradication of the underlying infection, debridement and decompression of the spinal canal in the presence of neurological deficits or epidural abscesses, and fixation of the affected segment to preserve or restore the spinal structure and stability. Long-term parenteral antibiotic therapy and immobilization dramatically increase the cost of care while significantly affecting the quality of life of patients [2,8].

Hyperbaric oxygen (HBO_2_) therapy is a treatment modality in which patients breathe 100% oxygen in a hyperbaric chamber that has been pressurized to greater than atmospheric pressure (1 atmosphere absolute, ATA). In accordance with Dalton and Henry’s laws, the amount of oxygen dissolved in plasma increases as the fraction of inhaled oxygen (FiO_2_) and pressure increases. HBO_2_ increases the partial pressure of oxygen (pO_2_) in both the local soft tissue and osteomyelitic bone. These high tissue pO_2_ levels restore the functions of neutrophils, fibroblasts, macrophages, osteoclasts, and osteoblasts that are deficient in specific anti-infectious, inflammatory, or reparative functions in environments where oxygen levels are insufficient, as in infected tissue. HBO_2_ also augments the bactericidal activity of certain antibiotics [9,10,11,12]. There are several published reports noting that HBO_2_ therapy is beneficial in the treatment of spinal infections [13,14,15,16]. However, there have been no randomized controlled studies examining the treatment indications and efficacy of HBO_2_ therapy in the management of these clinical entities. The aim of this study was to report our experience with HBO_2_ therapy in the treatment of pyogenic spinal infections in a retrospective review.

## 2. Material and Methods

We identified patients who were referred to our center for HBO_2_ therapy with a clinical diagnosis of spinal infections (vertebral osteomyelitis, pyogenic spondylitis, spondylodiscitis, surgical site infection following spine surgery, epidural abscess) through a retrospective search over a 10-year period between October 2006 and April 2016. The diagnosis of spinal infection in these patients was made on the basis of clinical, laboratory, and radiologic evaluations. The clinical data were extracted from the medical records and were reviewed for patient demographic characteristics (age, sex, and comorbidities), microbiologic evaluation, medical treatment received before HBO_2_ (duration, administration form), surgical intervention, HBO_2_ therapy received (number and duration of sessions, interval between onset of symptoms and HBO_2_) and final clinical outcome based on laboratory, clinical, and radiologic evaluations performed by the referred department.

Prior to HBO_2_, all patients were evaluated for contraindications such as the presence of untreated pneumothorax, radiologically indicated bullae or bleb, pregnancy, severe emphysema and chronic obstructive pulmonary disease (COPD) assessed by pulmonary function tests, uncontrolled seizure disorders, and cardiovascular instability. HBO_2_ therapy was administered in a multiplace hyperbaric chamber once a day for five days a week during the range of two to twelve weeks. The treatment pressure was 2.5 ATA and each session consisted of three 25-min oxygen periods with five-minute air breaks to reduce the risk of oxygen toxicity. The HBO_2_ sessions were conducted by trained healthcare personnel under the supervision of the hyperbaric medicine specialist. The decision to terminate HBO_2_ was given by the referral clinic. 

## 3. Results

A total of 19 patients with pyogenic spinal infection were referred for HBO_2_ therapy over a 10-year period. HBO_2_ was evaluated as contraindicated after consultation with the pulmonary diseases clinic for two patients: One patient had bullae in the lungs (who also had repeated *Candida albicans* proliferations in wound site cultures) and the other had subpleural air entrapment lesions with a history of tuberculosis, as shown by high-resolution computed tomography. In one patient, non-small cell lung cancer was detected, and he was referred so that his treatment could be continued by oncology and thoracic surgery clinics. Three patients discontinued treatment because of problems encountered during transport. Hence, the evaluations were made for 13 patients. The clinical characteristics of the patients are shown in Table 1.

**Figure 1:**
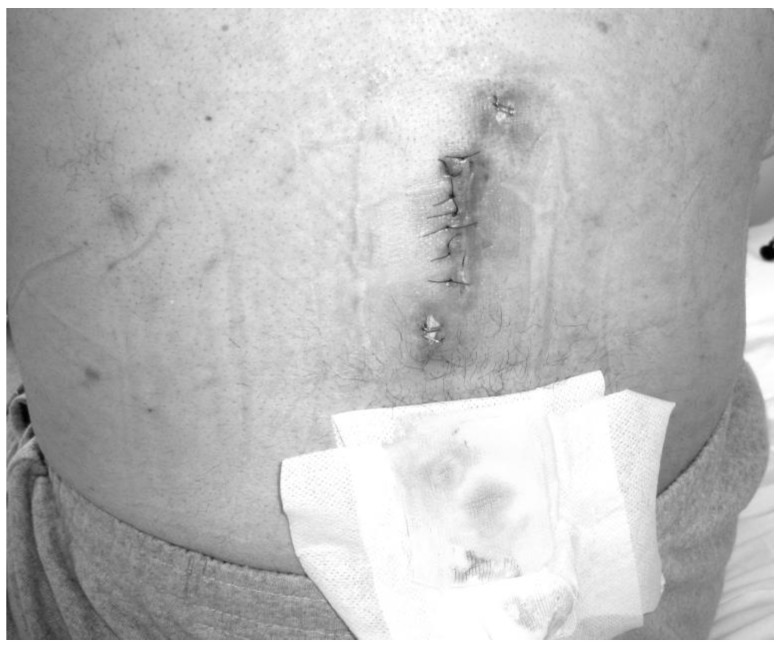
The appearance of the wound with purulent drainage in the operation site prior to HBO_2_ treatment.

**Figure 2:**
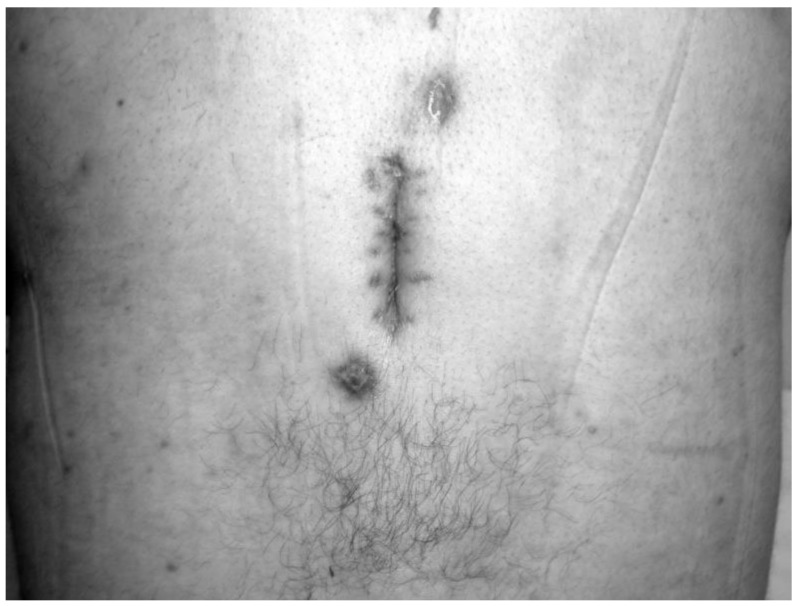
Discharge from the wound site stopped and complete wound healing was achieved at the end of HBO_2_.

All patients who received HBO_2_ treatment (seven males, six females) had a prior history of spinal surgery. The primary diagnosis was spondylolisthesis in one patient, fracture in two patients, stenosis in three patients, and disc herniation in seven patients. The mean time between diagnosis and HBO_2_ treatment was 7.8 weeks. In patients referred after 2012, however, this time was 5.2 weeks. The primary symptom of all patients was pain. On admission, five patients (38%) were able to stand and walk without assistance, three patients (23%) were able to stand and walk with assistance, and five patients (38%) were unable to stand and walk because of severe pain. Conservative orthopedic treatment consisted of immobilization with rigid orthosis (hard cervical collar, thoracolumbar rigid brace, lumbosacral corset), which continued until complete healing of infection. There was also a discharging wound or sinus in the operation site in five patients. A daily wound dressing was applied in this group. In four of these five patients (80%), complete wound healing was achieved at the end of HBO_2_ therapy (Figure 1 and Figure 2).

An appropriate antimicrobial regimen was chosen in all patients based on microbial sensitivity results and the recommendations of infectious disease consultants. Patients who were culture-negative received empiric broad-spectrum antibiotic therapy. It was not possible to obtain samples for culture from two patients because of the localization of infection. The results of the majority of positive cultures were staphylococci (83.3%). The median value for the period of intravenous antibiotic therapy was 42 days, which was usually followed by oral antibiotics until clinical improvement and normalization of laboratory findings (Table 2). Glycopeptides (vancomycin and teicoplanin) were the most frequently used intravenous antibiotics, and fluoroquinolones, clindamycin, and fusidic acid were the antibiotics chosen for oral therapy. Prior to HBO_2_ therapy, two patients were treated with incision and drainage for epidural abscess; two patients underwent irrigation, debridement, and hardware/pedicle screw removal; and one patient had incision, drainage, and underwent revision of spinal instrumentation.

**Figure 3:**
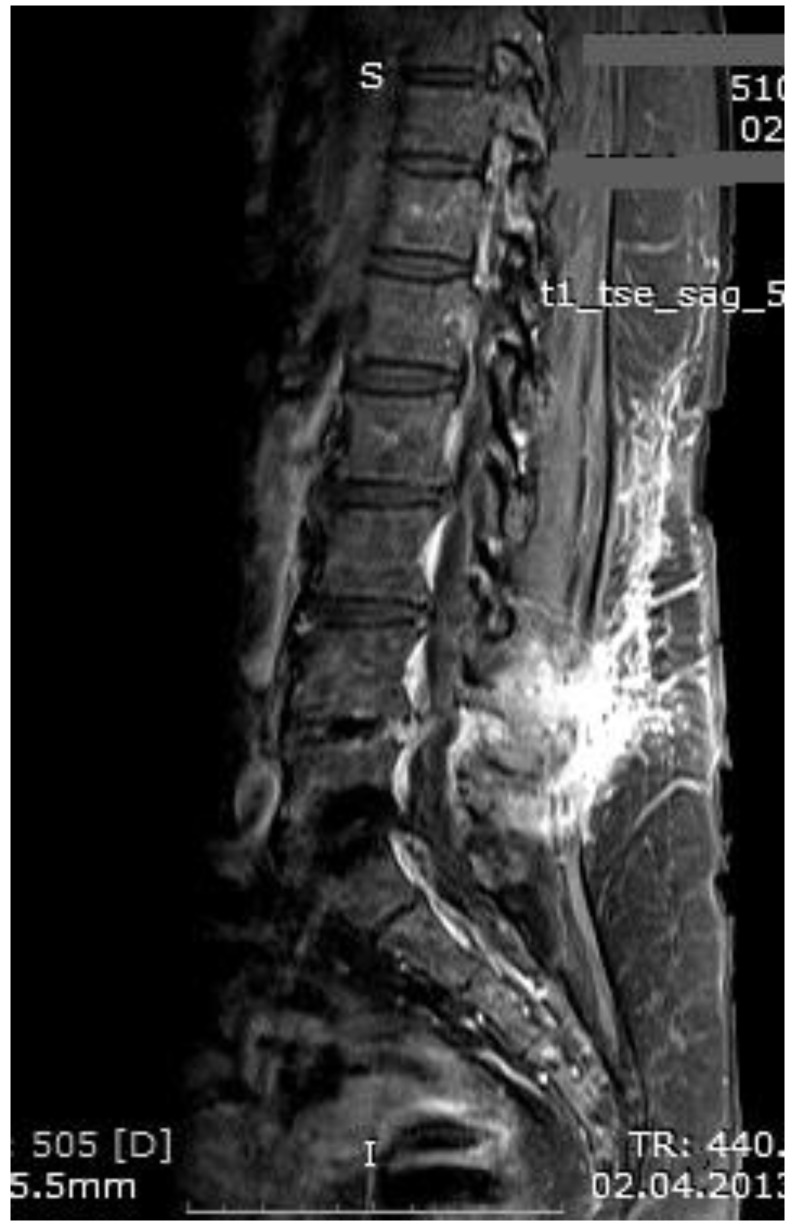
MR imaging T1-weighted scan showing inflammatory infiltrates in the course of pre-HBO_2_ treatment.

**Figure 4:**
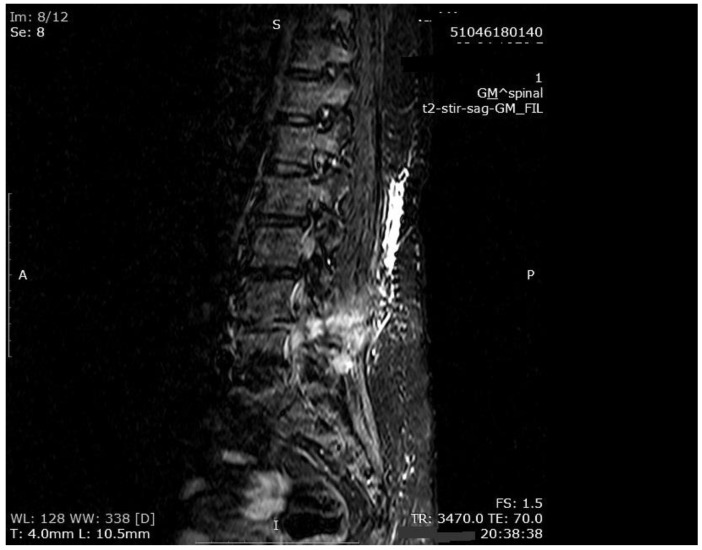
MR imaging T2-weighted scan showing inflammatory infiltrates in the course of pre-HBO_2_ treatment.

**Figure 5:**
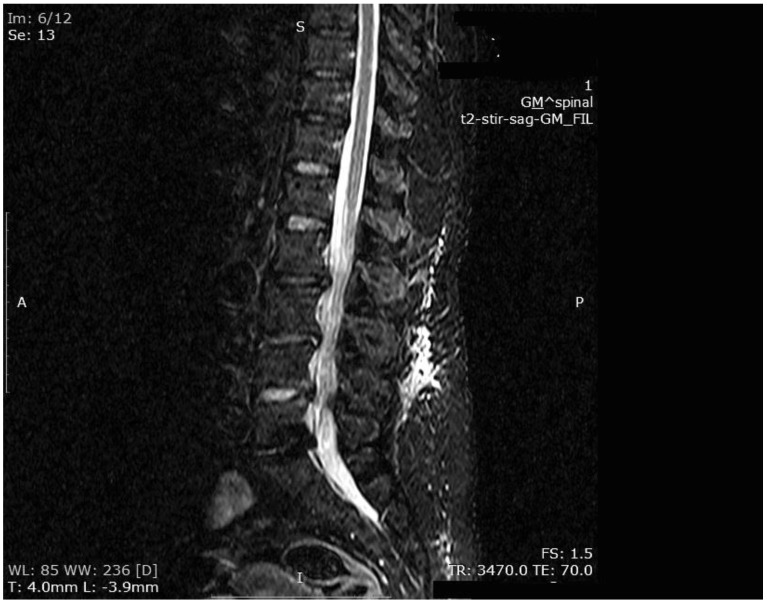
MR imaging scan showing inflammatory infiltrates at the end of HBO_2_ treatment.

The mean number of total HBO_2_ sessions was 33 (±18.5) sessions. The HBO_2_ treatment was tolerated well by all patients except two. One patient had to receive analgesics (non-steroidal anti-inflammatories) before HBO_2_ sessions because she had severe back pain, and in one patient the treatment was interrupted for five days because of minor middle ear barotrauma. Improvements in clinical evaluations and laboratory findings were observed in all cases at the end of the HBO_2_ therapy. The mean erythrocyte sedimentation rate (ESR) value reduced from 76.1 (range, 6–129) to 45.7 (range, 5–81) mm/h, and the mean C-reactive protein (CRP) concentration reduced from 82.9 (range, 14–202) to 17.5 (range, 1–49.5) mg/L (normal limits; ESR: <20 mm/h, CRP: 0–5 mg/L). Infection resolution was adequately achieved in 12 of 13 patients on MR imaging at the end of HBO_2_ treatment or during the first month of follow-up (Figure 3, Figure 4 and Figure 5). There was no significant improvement in one patient. To extend the total clinical follow-up period up to three years (mean: 11 months; range, 1 month–3 years), telephone interviews were conducted to determine any recurrence of symptomatic clinical infection. No recurrence of infection, spinal instability, or deformity was seen in any patients.

## 4. Discussion

This retrospective clinical study suggests that HBO_2_ therapy is efficacious in patients with pyogenic spinal infections complicated by primary therapy failure or by medical comorbidities that may impede the eradication of microbial infection and delay wound healing. Pyogenic infections of the spine are a heterogeneous group of disorders. The confusion in the nomenclature, particularly in intervertebral disc space involvement, is hidden in the details of the pathophysiology [2,17]. Contrary to infantile discs, the intraosseous arterial anastomoses of adult intervertebral discs undergo an involution around the third decade of life, creating the largest avascular structure in the human body [18]. Three different routes of pathogen spread are classic hematogenous, direct external inoculation, and spread from adjacent tissues. The involvement of the disc space and two adjacent vertebral bodies is described as spondylodiscitis, which has classic imaging of erosion of vertebral endplates, osteolytic lesions, and compression fractures, and can lead to spine instability, deformity, and risk of spinal cord compression [2,17]. It has been suggested that primary spondylodiscitis should be considered as a distinct entity from vertebral osteomyelitis, and that these lesions behave differently than vertebral osteomyelitis. In essence, the controversy about the pathogenesis of primary spondylodiscitis and its association with vertebral osteomyelitis is related to whether the adult disc can be seeded hematogenously. An increasing number of studies performed to clarify the pathophysiology of intervertebral disc degeneration also elucidated the pathophysiology of spondylodiscitis while responding to these discussions. In this context, there has been little controversy with regard to pathogenesis of postprocedural or iatrogenic discitis ever since the first description by Turnbull [2,3,19,20,21,22]. The patients’ clinical characteristics in our study were consistent with this description due to the history of previous surgical interventions in all patients who were referred for HBO_2_ therapy. The exception is one patient who was diagnosed as having infective endocarditis. Further spread of infection beyond the bone structures can access the surrounding tissues, causing paravertebral and psoas abscesses. When spreading into the spinal canal, it can cause epidural abscesses, subdural abscesses, and meningitis [2,8,17]. The devastating outcomes for the patient and the major costs of spinal infections to the healthcare system are due to prolonged hospitalization, increased patient care, and additional surgical interventions such as necrotic tissue debridement, irrigation, abscess draining, fusion, and hardware revision or removal. The fixation material, however, cannot be removed as easily because of instability. Hence, any treatment that could improve outcomes and reduce the need for reoperations would be extremely valuable [13,15].

The underlying rationale for the use of HBO_2_ therapy as an effective adjuvant in the treatment of pyogenic spinal infections is the mechanism of action overlap with the pathophysiologic process. When there is a direct inoculation to the disc space—especially in the presence of disc degeneration or a traumatic condition, which means that the annulus fibrosis and nucleus pulposus are infiltrated by new vascular formations—the acute inflammation progresses much faster [23]. The ischemia-induced endogenous lysosomal activity of neutrophils destroys the disc and adjacent endplate. When the endplates are breached, particularly in the presence of comorbidities, the infecting organisms may spread to the adjacent vertebral bodies and cause bone destruction and collapse. Avascular necrosis, bone infarction, and abscess formation can develop rapidly in this suppurative and ischemic background [21,22,23,24]. Under ambient conditions, pO_2_ in infected bone is lower than normal bone, which is secondary to this hypoperfusion and inflammation. The inflammatory process increases oxygen consumption, mainly due to increased consumption by polymorphonuclear leukocytes. It has been shown that *Staphyloccus epidermidis*, *Escherichia coli*, *Pseudomonas aeruginosa*, as well as *Staphyloccus aureus* are inefficiently killed by phagocytes while under hypoxic conditions as compared with their activity under HBO_2_ tension. Furthermore, increased pO_2_ is directly lethal to anaerobic and some microaerophilic organisms that lack the ability to produce oxygen radical-scavenging enzymes such as superoxide dismutase and catalase [9,11,15,23,24]. In the subacute and early regenerative phases, granulation tissue fills the void caused by the destruction, and new bone forms over time, [23,24]. The low oxygen levels in infected bone result in slow bone healing due to the inhibition of fibroblast, osteoclast, osteoblast, and macrophage activity. Resolution of infection, as well as bone healing, are often impeded in the presence of comorbidities such as diabetes mellitus, peripheral vascular disease, obesity, and concomitant degenerative bone disease, malignancy, radiation damage or chemotherapy. Recurrent surgery compromising grafts or flaps, and foreign material, can make the condition much more complicated. HBO_2_ helps provide adequate oxygen for osteoclastic, osteoblastic, and fibroblastic activity for bone repair, angiogenesis, and wound healing in hypoperfused, hypoxic, and infected tissues [9,13,15]. 

Although the optimal duration remains controversial, the standard approach to treating spinal infections includes administration of microbial sensitivity-guided intravenous antibiotics for at least four to six weeks. With extensive spread into paraspinal soft tissues and undrained psoas muscle abscesses, therapy may be extended to up to eight weeks [2,8,13,17]. The synergistic effect of HBO_2_ therapy, when used in combination with antibiotic therapy in the treatment of osteomyelitis, has been reported in several studies [9,10,25]. This effect depends on the penetrating ability of systemically administered antibiotics, as well as the characteristics of the infected area to which they penetrate. Using *P. Aeruginosa*, Mader et al. demonstrated that the bactericidal activity of the aminoglycoside class of antibiotics was enhanced when oxygen tension was elevated above hypoxic levels [9,10]. However, the situation seems much more complicated for adult discs because they have no direct blood supply; their nourishment occurs via the capillaries in the outer annulus fibrosus, or by diffusion through endplates, which act as a selectively permeable barrier to solutes. Systemic antibiotics also need to enter the disc in the same way. Therefore, the properties of antibiotics such as size, solubility, binding, and charge, in particular, have been widely discussed in the literature [22,26]. It has been postulated that negatively charged antibiotics (e.g., penicillins, cephalosporins) have limited penetration because the proteoglycan concentration is high in the nucleus pulposus, whereas positively charged antibiotics (e.g., aminoglycosides, glycopeptides, and clindamycin) have adequate penetration [27,28]. In contrast, Walters et al. suggested that cephazolin, which is negatively charged and the most frequently used antibiotic, was able to penetrate the disc in humans and animal models when used prophylactically [21,22]. Zhang et al. also demonstrated that cephazolin and clindamycin could penetrate into infected and normal nucleus pulposus, and the penetration rate was associated with the presence of infection as well as charge characteristics [26]. However, to our knowledge, there is no systematic empiric research addressing the question of how HBO_2_ therapy affects the penetration of antibiotics into intervertebral discs.

Another purpose of this study was to evaluate how HBO_2_ treatment was tolerated in this patient group. HBO_2_ therapy is regarded as a relatively benign intervention [12,29,30]. Middle ear barotrauma is the most common adverse effect of HBO_2_. It can be prevented by teaching patients how to equalize pressure during compression. Equalization is possible with the active effort of the patients themselves. It may be difficult with patients who have limited capacity for active opening of the eustachian tubes, such as babies, children, patients under sedation or mechanical ventilation, or those with an inflammatory condition in the nasopharynx caused by viral infection, allergy, or gastroesophageal reflux [29,30]. In addition, attention must be paid to a patient’s position, particularly during the compression phase. It is well known that the supine position results in increased central venous pressure, and leads to venous congestion, making it more difficult to equalize middle ear pressure [29]. Almost all of our patients were admitted for treatment in the supine position due to ongoing immobilization treatments and/or pain. However, the prevalence of middle-ear barotrauma was lower than expected in our study. This can be attributed to several factors including slow compression procedures, switching a patient’s position to sitting (Fowler) or semi-sitting (Semi-Fowler) during compression, the attendance of an experienced hyperbaric nurse, and teaching patients self-inflation maneuvers during otoscopic examinations prior to sessions. Transport is one of the fundamental aspects that is often overlooked in the treatment of this patient group. The major problem affecting the treatment continuum is due to the intensification of pain during the transfer of patients between facilities. Hence, three patients did not continue therapy after one to three sessions.

The optimum timing of HBO_2_ therapy for pyogenic spinal infections remains unclear. In our series, the HBO_2_-treated patients comprised those whose iatrogenic spinal infections persisted despite the treatments applied and showed no improvement in laboratory findings. It may be thought that HBO_2_ could be started earlier when the pathogenesis of the disease is taken into consideration. However, further evidence is needed to make a clear statement. The appropriate duration of HBO_2_ therapy for pyogenic spinal infections also remains controversial. Reported treatment regimens for various series typically vary from between 10–40 sessions in total [13,14,15,16]. It has been suggested that the establishment of the healing process and infection control can be achieved in a short time. However, to achieve the bone remodeling phase and long-term infection control, longer treatment protocols are required (e.g., up to or more than 40 sessions) [15]. In our series, the number of HBO_2_ sessions applied was determined by the neurosurgery and infectious disease clinics based on the individual responses to treatment.

There are several limitations in the present study. First, because of its retrospective design, it does not include an approach that enables comparisons of the outcomes of patients treated with antibiotic therapy, immobilization, and surgical intervention, or those who received adjuvant HBO_2_ therapy. Secondly, the patients included in our study represented a much more complicated subgroup of pyogenic spinal infections because of the primary treatment failure, or because of the presence of several medical comorbidities that could impede the eradication of microbial infection. Finally, our analysis was based on a limited number of patients, and the small sample size did not allow an evaluation of the influence of these comorbidities on the outcome. Despite the limitations mentioned above, this study still provides clinical value. HBO_2_ therapy may be particularly useful for patients with primary treatment resistance and comorbidities, and could help to reduce long hospital stays, repeated surgeries, and morbidities. Awareness of this information will help to decrease the costs of pyogenic spinal infections to patients and the healthcare system.

## 5. Conclusions

This study suggests that HBO_2_ therapy is efficacious in patients with pyogenic spinal infections complicated by primary therapy failure or by medical comorbidities that may impede the eradication of microbial infection and delay wound healing. HBO_2_ therapy may be useful at reducing long hospital stays, repeated surgeries, and morbidities.

## Figures and Tables

**Table 1:** Summary of clinical characteristics of 19 patients who referred to HBO_2_ therapy.

Age, Mean (Years)	57 (Range: 36–78)
Sex	*n*
Male	9 (47.3%)
Female	10 (52.6%)
Diagnosis before spinal surgery	
Disk hernia	9 (47.3%)
Disk hernia, spondylolisthesis	1 (5.2%)
Vertebral fracture	3 (15.7%)
Spondylolisthesis	2 (10.5%)
Stenosis	3 (15.7%)
No prior history of spinal intervention	1 (5.2%)
Interval between onset of symptoms and diagnosis	
≤4 weeks	10 (52.6%)
>4 weeks	9 (47.3%)
Comorbidities and risk factors	
Diabetes mellitus	8 (42.1%)
Hypertension	10 (52.6%)
Ischemic heart disease	4 (21.0%)
Infective endocarditis	1 (5.2%)
Smoking	11 (57.8%)
Alcohol use	1 (5.2%)
Prior history of malignancies	3 (15.7%)
Prior history of tuberculosis	1 (5.2%)
Chronic anemia	1 (5.2%)
Hypothyroidism	2 (10.5%)
Peptic ulcer disease	1 (5.2%)
No risk factors or comorbidities	3 (15.7%)
Localization	
Cervical	1 (5.2%)
Thoracic	1 (5.2%)
Thoracolumbar	1 (5.2%)
Lumbar	11 (57.8%)
Lumbosacral	5 (26.3%)
Presence of adjacent tissues involvement (adjoining soft tissue, medullary canal)	16 (84.2%)
Abscess formation	
Paravertebral	11 (57.8%)
Postvertebral (epidural)	5 (26.3%)
Arachnoiditis	2 (10.5%)
Microbiological evaluation	
Number of culture-positive cases	9 (47.3%)
Isolated microorganisms	
*Methicillin-sensitive Staphylococcus aureus* (MSSA)	3 (15.7%)
*Staphylococcus epidermidis*	2 (10.5%)
*Methicillin-resistant Staphylococcus aureus* (MRSA)	1 (5.5%)
*Pseudomonas aeruginosa*	1 (5.2%)
*Escherichia coli*	1 (5.2%)
*Candida albicans*	1 (5.2%)
Number of culture-negative cases	8 (42.1%)
Unachievable obtaining of the sample for culture	2 (10.5%)
Biochemical parameters	
Erythrocyte sedimentation rate (ESR), mm/h (mean ± standard deviation, SD)	76.1 ± 31.4
Elevated (>20 mm/h)	18 (94.7%)
White blood cell (WBC) count, 10^9^/L (mean ± SD)	10.47 ± 4.2
Elevated (>9.0 × 10^9^/L)	11 (57.8%)
C-reactive protein (CRP), mg/L (mean ± SD)	80.9 ± 60.4
Elevated (>10 mg/L)	19 (100%)

(*n*, number of patients).

**Table 2:** Summary of clinical characteristics of patients with postoperative pyogenic spinal infection who underwent HBO_2_ therapy (n*, number of patients).

Interval between spinal surgery and diagnosis of spinal infection	n*
≤4 weeks	4 (30.7%)
>4 weeks	9 (69.2%)
Interval between diagnosis of spinal infection and onset of HBO_2_ therapy	
≤4 weeks	6 (46.1%)
>4 weeks	7 (53.8%)
Number of treatment sessions (mean ± standard deviation, SD)	33.2 ± 18.5
Intravenous antibiotic therapy Glycopeptide + beta-lactam	7 (53.8%)
Beta-lactam monotherapy	5 (38.4%)
Glycopeptide monotherapy	1 (7.6%)
Duration of intravenous antibiotic therapy (days) (mean ± SD)	42.8 ± 13.1
Biochemical parameters	
Erythrocyte sedimentation rate (ESR)	
Before HBO_2_, mm/h (mean ± SD)	76.2 ± 34.8
After HBO_2_, mm/h (mean ± SD)	45.7 ± 30.3
WBC count	
Before HBO_2_, 10^9^/L (mean ± SD)	9.29 ± 3.49
After HBO_2_, 10^9^/L (mean ± SD)	7.31 ± 1.91
C-reactive protein (CRP)	
Before HBO_2_, mg/L (mean ± SD)	82.9 ± 58.3
After HBO_2_, mg/L (mean ± SD)	17.5 ± 17.7
Response to HBO_2_ (Outcomes of MR imaging after HBO_2_ therapy)	
Infection resolution	12 (92.3%)
No significant improvement	1 (7.6%)

n*, number of patients.
